# ASO Author Reflections: the Role of Dispositional Optimism for Better Health-Related Quality of Life after Esophageal Cancer Surgery

**DOI:** 10.1245/s10434-021-10055-5

**Published:** 2021-04-26

**Authors:** Yangjun Liu, Pernilla Lagergren

**Affiliations:** 1 Department of Molecular Medicine and Surgery, Karolinska Institutet, Karolinska University Hospital, Stockholm, Sweden; 2 Department of Surgery and Cancer, Imperial College London, London, UK

## Past

As the mainstay of curative treatment for esophageal cancer, surgery increases survival but entails impaired health-related quality of life (HRQL).^1^ To improve postoperative HRQL, it is important to identify its predictors. The already identified sociodemographic and clinical predictors cannot fully explain the variation of HRQL after esophageal cancer surgery, suggesting that other factors may also play a role. Dispositional optimism, a personality trait, has been found to be associated with HRQL in patients with other subtypes of cancer.^2^,^3^ However, no such studies have been conducted in patients with esophageal cancer.

## Present

The current Swedish nationwide longitudinal study^4^ assessed whether higher dispositional optimism predicted better HRQL after esophageal cancer surgery. Patients in this study were categorized into four groups with hierarchical dispositional optimism levels. Postoperative HRQL was measured using the European Organization for Research and Treatment of Cancer (EORTC) Quality of Life Questionnaire-Core 30 (QLQ-C30) and Quality of Life Questionnaire-Esophago-Gastric module 25 (QLQ-OG25). This study found that patients with higher dispositional optimism reported better global quality of life, emotional function, social function, and self-perceived body image, and fewer self-reported problems in pain, dyspnea, diarrhea, anxiety, dry mouth, trouble with taste, and worry about weight loss after esophageal cancer surgery. The predictive effect of dispositional optimism may help identify patients with high risk of suffering from poor HRQL recovery after esophageal cancer surgery, and thus may lead to timely and tailored interventions to improve postoperative HRQL.

## Future

Studies elucidating potential effective interventions to improve postoperative HRQL are warranted. It might be worth examining whether increasing dispositional optimism could improve postoperative HRQL. Identifying the potential modifiable mediators between dispositional optimism and HRQL may also be valuable because some potential mediators such as coping strategy and social support may be easier to be modified than dispositional optimism. In addition, interventions to prevent or relieve psychological distress may also help improve HRQL after esophageal cancer surgery.^5^
